# Botulinum Neurotoxin Injections in Childhood Opisthotonus

**DOI:** 10.3390/toxins13020137

**Published:** 2021-02-12

**Authors:** Mariam Hull, Mered Parnes, Joseph Jankovic

**Affiliations:** 1 Section of Pediatric Neurology and Developmental Neuroscience, Texas Children’s Hospital and Baylor College of Medicine, Houston, TX 77030, USA; parnes@bcm.edu; 2 Parkinson’s Disease Center and Movement Disorders Clinic, Department of Neurology, Baylor College of Medicine, Houston, TX 77030, USA; josephj@bcm.edu

**Keywords:** opisthotonus, opisthotonos, axial dystonia, botulinum toxin

## Abstract

Opisthotonus refers to abnormal axial extension and arching of the trunk produced by excessive contractions of the paraspinal muscles. In childhood, the abnormal posture is most often related to dystonia in the setting of hypoxic injury or a number of other acquired and genetic etiologies. The condition is often painful, interferes with ambulation and quality of life, and is challenging to treat. Therapeutic options include oral benzodiazepines, oral and intrathecal baclofen, botulinum neurotoxin injections, and deep brain stimulation. Management of opisthotonus within the pediatric population has not been systematically reviewed. Here, we describe a series of seven children who presented to our institution with opisthotonus in whom symptom relief was achieved following administration of botulinum neurotoxin injections.

## 1. Introduction

**Figure 1:**
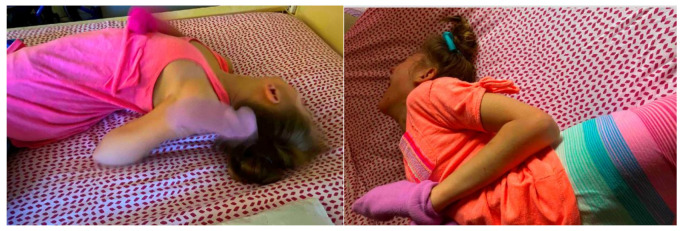
Illustrative photographs of opisthotonus of patient 4 prior to receiving treatment displaying characteristic axial extension of the trunk and neck.

Opisthotonus, derived from the Greek “opistho” meaning behind and “tonos” meaning tone, was described by Nayler in 1803 within the context of tetanus as “such rigid contractions of those of the back, as to draw his head forcibly backwards at the same that his chest was considerably elevated” and described his expression as “bearing the strongest possible marks of the agony he suffered” [1]. Later, it was described by Charcot in 1887 as “arc de cercle”, or arching of the body such that the patient is suspended above the bed by the head and the heels [2]. Charcot’s description of abnormal posture was in the context of hysteroepileptic spells, later classified as psychogenic or functional dystonia [3,4]. Opisthotonus involves forceful axial extension of the trunk and also neck (retrocollis), caused by involuntary contractions of the paraspinal muscles, often associated with dystonic posturing of arms and legs (Figure 1) [5]. Although most frequently attributed to dystonia, there are many causes of opisthotonus that have been identified (Table 1). While in adults, the most common cause of opisthotonus is tardive dystonia [6], typically associated with adduction of the shoulders and extension/pronation of the arms, the most common causes of childhood opisthotonus are perinatal hypoxic-ischemic injury, and genetic/metabolic disorders which often present as a cerebral palsy phenotype [5].

Opisthotonus represents one of several abnormal axial skeletal abnormalities en-countered in adult and pediatric populations, also including camptocormia, scoliosis, and other postural abnormalities [7,8]. It can be persistent and severe, occurring in-termittently or for sustained periods throughout the day, and when it presents as such, it is incompatible with living comfortably at home. If left untreated, many patients with these axial abnormalities develop contractures, fixed deformities due to shorten-ing of muscles and tendons. In some cases of dystonic opisthotonus, the intense con-tractions of the paraspinal muscles can lead to dystonic storm (also known as status dystonicus) [9,10]. This potentially fatal condition is characterized by persistent, worsening dystonia associated with interference with sleep as well at least one meta-bolic disturbance: (1) fever not due to infectious etiology, (2) abnormal electrolytes, (3) elevated CK ≥ 1000, (4) myoglobinuria [10]. Although relatively rare, dystonic storm is a life-threatening emergency and its complications can lead to respiratory failure, re-nal failure, or even death [10,11].

**Table 1:** Etiologies of opisthotonus.

Acquired	Genetic/Metabolic
*Hypoxic injury*	Krabbe disease [12]
Perinatal asphyxia [5]	Gaucher disease [13]
Near-drowning [5]	Adenylosuccinate lyase deficiency [14]
Cardiac/respiratory arrest [2]	Lesch-Nyhan syndrome [15]
Traumatic asphyxia [2]	Glutaric aciduria [16]
*Infectious/Post-infectious*	Maple syrup urine disease [17]
Meningitis [18,19]	Monoamine neurotransmitter defect [20,21]
Encephalitis [22,23]	Wilson disease [24]
Tetanus [1,25,26,27,28]	Neurodegeneration with brain iron accumulation (NBIA), e.g., pantothenate kinase-associated neurodegeneration (PKAN) [15]
Rabies [29]	Other genetic disorders ^1^ [21,30]
Neurosyphilis [31]	
Cerebral malaria [12]	
*Toxins/elevated toxic metabolites*	
Kernicterus [12]	
Strychnine [32]	
Phencyclidine [32]	
Phenothiazines [32]	
Propofol [33,34]	
Methoxphenidine [35]	
Neonatal caffeine overdose [32]	
*Increased Intracranial Pressure*	
Subarachnoid hemorrhage [5]	
Intracerebral hemorrhage [5]	
Hydrocephalus [36]	
Mass/Tumor [36]	
*Autoimmune*	
Stiff-person syndrome [37]	
Anti-NMDA receptor encephalitis [22]	
*Other*	
Functional (psychogenic) dystonia [3,4,38]	
Tardive dystonia [6,30,39,40,41,42,43]	
Malignant catatonia [29]	
Brainstem/cerebellar malformation [44]	
Epilepsy [12]	

^1^ Any genetic syndromes with prominent dystonia have the potential to manifest opisthotonus.

Treatment of opisthotonus is multifactorial, first targeting reversible causes such as discontinuation of the offending dopamine-receptor blocking drugs that may be causing acute or tardive dystonia, and the consideration of disease-specific therapies. In most cases, no such interventions are available, and supportive measures are employed, often consisting of a tiered approach beginning with oral medications (benzodiazepines, baclofen, and anticholinergics), and in more refractory cases requiring intravenous benzodiazepines for exacerbations and/or surgical interventions such as intrathecal baclofen pump or deep brain stimulation [45,46,47].

Over the past half-century, botulinum neurotoxin (BoNT) has emerged as one of the most versatile therapeutics in neurologic and non-neurologic disorders [48]. There are dozens of indications in the field of movement disorders alone [49], including treatment of spasticity and a variety of abnormal involuntary movements such as tremor, tics, hemifacial spasm, and most commonly focal/segmental dystonia [50]. In children with refractory generalized dystonia, areas of the body that are dispropor-tionately affected, or in which the dystonia causes a great deal of discomfort, can also be selectively targeted with BoNT. It acts primarily by inhibiting the release of acetyl-choline from the presynaptic nerve terminal through interfering with fusion of the synaptic vesicle with the plasma membrane at the level of the neuromuscular junction.

Here, we describe a series of children suffering from opisthotonus whose symptoms were adequately controlled using BoNT injections.

## 2. Results

We evaluated seven patients (four female), age range 15 months to 13 years, all with opisthotonus, who were treated at our institution with BoNT (Table 2). Etiologies varied, with three due to identified genetic causes including pantothenate kinase asso-ciated neurodegeneration (PKAN), hypomyelinating leukodystrophy-14, CASK-related disorder, and a single case with undetermined, likely genetic etiology with normal im-aging and lack of other risk factors. Three of the patients presented with dystonic storm.

All patients were also treated with oral clonazepam and baclofen with partial though insufficient benefit. Two patients received trials of oral clonidine which was limited by excessive somnolence and hypotension as well as no clear improvement in opisthotonus, and therefore discontinued in both patients. One patient had presumed tardive dystonia and akathisia and was also treated with tetrabenazine, which was helpful for the akathisia, but with no clear improvement in the axial dystonia. The three patients who presented with dystonic storm were also managed with intrave-nous sedation, which led to control of symptoms until attempting to wean the intra-venous therapies, at which time posturing worsened.

Sites and dosing were chosen based on the involved muscles and estimated muscle mass and force of muscle contraction. All patients received onabotulinumtoxinA injec-tions in the paraspinal muscles and six patients received injections at other sites in-volved, with the most common being splenius capitis for management of concurrent retrocollis (five out of seven patients) (Table 3). Dosing to the paraspinal muscles ranged between 120 to 300 units (average 215.7 units) and were divided into three to five injections on each side. Total units administered ranged between 150 to 650 units (average 341.4 units). Although the dosing regimen was not planned using weight-based calculations, total units administered per kilogram for these patients ranged from 16.7 to 23.8 U/kg (average 19.6 U/kg).

All patients showed an improvement in opisthotonus within 3–14 days (average 6.1 days). Arching improved notably on examination of each child, and improvement was deemed to be of a meaningful and sufficient degree by caregivers of all injected children. There were no adverse effects with the exception of one patient who experi-enced neck extensor weakness, which resolved over a period of weeks and did not re-cur with subsequent injections omitting the splenius capitis. Four of seven patients re-quired repeat injections approximately three months following initial injections. No patient in this series has required escalation of treatment to involve surgical manage-ment of tone thus far. See Video S1 and Video S2 for illustrative cases before and after treatment.

## 3. Discussion

This is the first report of childhood opisthotonus successfully treated with BoNT injections. The first description of adequate treatment of opisthotonus was by Nayler in 1803 in which he treated a 36-year-old man suffering from presumed tetanus fol-lowing a farming injury. Nayler quickly began treatment of the man which included “bladders of warm water to his feet, forty drops of laudanum to be taken every four hours in a camphorated mixture with tincture of castor.” When there was no im-provement, he also ordered “wine freely, to the amount of a bottle in the day with a strong decoction of the bark and valerian, at the same time a liniment well charged with opium was rubbed into his chest and limbs twice a day” [1]. After some period of time had passed, the patient’s symptoms eventually improved.

In 1877, another patient was reported with opisthotonus, as a result of an empy-ema following a traumatic brain injury. He was treated with “a mixture containing eighty grains of chloral-hydrate, half a drachm of extractum opii liquidum, and cam-phor” [51].

Since then, the approach to treatment of opisthotonus has been largely empirical and unsuccessful, typically involving a trial of several oral medications [30]. It was not until 1991, when Narayan and colleagues treated a patient with severe opisthotonus and generalized dystonia suffering from dystonic storm with urgent intrathecal baclo-fen that the use of more invasive measures became more common [47].

Further reports of surgical management of opisthotonus include deep brain stim-ulation [46] and intrathecal baclofen [5,13,47], each with clinical benefit. However, not all patients are good surgical candidates, the procedures are not without risks, both in-traoperatively and long-term, and there can be insufficient benefit from either surgical treatment.

Our series of patients provides evidence that there is a role for BoNT injections in combination with pharmacotherapy, and prior to or supplemental to surgical inter-ventions, in children with axial dystonia, including opisthotonus. Retrospective studies have reported doses of up to 15–20 U/kg, and total doses of 800–1200 U have been safe; however, there have not been any large prospective studies in children determining a true upper dose limit [52,53,54,55]. Furthermore, the treatment was well-tolerated within this group, with no serious adverse events, and only a single case of transient neck ex-tensor weakness. None of the seven patients treated with injections required surgical treatment.

The three patients who did not require repeat injections in the months immedi-ately following initial injections may not have needed them due to the fluctuating na-ture of their underlying dystonia. This was evidenced by the eventual decrease in pos-turing even in sites not injected. Other potential reasons for improvement of dystonia also include management of pain, constipation, underlying infection, or other potential triggers of worsening dystonia, as well as maturing neural networks within the young-er population. Repeat injections remain an option, should dystonia become exacerbat-ed again in the future.

Limitations of this study include its retrospective, open-label design. Injections were performed using anatomic landmarks, without electromyogram or ultrasound guidance. Moreover, there is no objective scoring system that is clearly applicable to the pediatric population, though by subjective report, video (Video S1, Video S2), and clinical documentation, there was clear improvement of opisthotonus in these patients.

## 4. Conclusions

Despite the retrospective, small, and open-label design, our study provides evidence that BoNT injections into paraspinal muscles ameliorates childhood opisthotonus without serious adverse effects. This treatment should be added to the armamentaria of treatment options for this form of axial dystonia, particularly in cases refractory to oral medications. Of note, over half of the patients reviewed here are believed to have a genetic etiology for their opisthotonus.

## 5. Materials and Methods 

Here, we describe seven patients who presented to our center for management of opisthotonus and were treated with BoNT injections. Patients were followed clinically and we discussed features, dosing regimen, and outcomes through a review of clinical documentation and laboratory and radiology reports.

## Figures and Tables

**Table 2:** Characteristics of patients undergoing treatment of opisthotonus.

Patient	Sex	Age	Genetic vs. Acquired Etiology of Opisthotonus	Weight (kg)	History of Dystonic Storm
1	M	15 mo	Acquired—Post-infectious	7.3	Yes
2	F	26 mo	Genetic—Hypomyelinating leukodystrophy-14	8.5	Yes
3	M	8 yr	Genetic—NBIA/PKAN	23.7	No
4	F	10 yr	Acquired—Perinatal HIE	25.7	Yes
5	F	3 yr	Genetic—presumed	14.7	No
6	M	26 mo	Genetic—*CASK-*related disorder	12.2	No
7	F	13 yr	Acquired—Tardive dystonia	34.4	No

M = male, F = female, NBIA = neurodegeneration with brain iron accumulation, PKAN = pantothenate kinase associated neurodegeneration, HIE = hypoxic-ischemic insult, *CASK* = calcium/calmodulin-dependent serine protein kinase gene.

**Table 3:** Botulinum neurotoxin dosing regimens for patients with prominent opisthotonus.

Patient	Paraspinal Dosing (Units)	Other Muscles Injected	Total Dose (Units)	Latency (Days)	Adverse Effects	Duration of Benefit (Weeks)	Outcome	Repeat Injections Required	Other Treatments ^1^
1	150	None	150	3	None	12	Complete resolution of opisthotonus during maximal effect	Yes	Baclofen, clonazepam, diazepam, lorazepam, clonidine
2	120	Splenius capitis	170	5	None	14	Complete resolution of opisthotonus during maximal effect	Yes	Clonazepam, midazolam, dexmedetomidine, baclofen
3	260	Triceps brachii, pectoralis major, gluteus maximus	400	14	None	14–16	Complete resolution of opisthotonus during maximal effect	Yes	Clonazepam, baclofen, tetrabenazine
4	300	Splenius capitis, sternocleidomastoid	430	6	None	12–16	Complete resolution of opisthotonus during maximal effect	No ^2^	Clonazepam, lorazepam, diazepam, baclofen, carbidopa/levodopa, gabapentin, dexmedetomidine, morphine
5	160	Splenius capitis, gastrocnemeus, rectus femoris	300	7	None	10	Complete resolution of opisthotonus during maximal effect	No ^3^	Clonazepam, baclofen, phenol injections
6	220	Splenius capitis	290	3	None	12–14	Complete resolution of opisthotonus during maximal effect with gradual wearing off, remaining dystonia not impairing	No	Clonazepam, baclofen
7	300	Splenius capitis, gastrocnemeus	650	5	Neck weakness ^2^	16	Complete resolution of opisthotonus during maximal effect	Yes	Clonazepam, lorazepam, diazepam, trihexyphenidyl, baclofen, haloperidol, tetrabenazine, guanfacine, clonidine, carbidopa/levodopa, propranolol, carbamazepine, topiramate, duloxetine, cyclobenzaprine, methocarbamol, morphine, ketamine

^1^ Includes treatments tried by previous providers/institutions. ^2^ Subsequent injections omitted splenius capitis with resolution of symptoms ^3^ Increased posturing after wearing off was managed with increased dose of oral clonazepam ^3^ and oral baclofen.

## Data Availability

The data presented in this study are available on request from the corresponding author. The data are not publicly available to maintain patient privacy.

## Supplementary Materials

The following are available online at https://www.mdpi.com/2072-6651/13/2/137/s1. Video S1: Patient 1 prior to botulinum neurotoxin injections and nine days following injections. Video S2: Patient 2 prior to botulinum neurotoxin injections and five days following injections.
